# Regenerative medicine for the kidney: stem cell prospects & challenges

**DOI:** 10.1186/2001-1326-2-11

**Published:** 2013-05-21

**Authors:** Yue Li, Rebecca A Wingert

**Affiliations:** 1 Department of Biological Sciences, University of Notre Dame, Notre Dame, IN 46556, USA

**Keywords:** Renal stem cell, Renal progenitor, Kidney regeneration, Acute kidney injury, Chronic kidney disease

## Abstract

The kidney has key roles in maintaining human health. There is an escalating medical crisis in nephrology as growing numbers of patients suffer from kidney diseases that culminate in organ failure. While dialysis and transplantation provide life-saving treatments, these therapies are rife with limitations and place significant burdens on patients and healthcare systems. It has become imperative to find alternative ways to treat existing kidney conditions and preemptive means to stave off renal dysfunction. The creation of innovative medical approaches that utilize stem cells has received growing research attention. In this review, we discuss the regenerative and maladaptive cellular responses that occur during acute and chronic kidney disease, the emerging evidence about renal stem cells, and some of the issues that lie ahead in bridging the gap between basic stem cell biology and regenerative medicine for the kidney.

## Introduction

The kidney performs essential physiological jobs ranging from metabolic waste excretion to homeostatic functions like osmoregulation. Kidney diseases are currently a global public health problem, with an incidence that has reached epidemic proportions and continues to climb in the U.S. and worldwide [1]. These trends correlate with the global rise in the aged population and the increasing prevalence of conditions that cause renal complications, namely cardiovascular disease, hypertension and diabetes [2]. Kidney diseases arise from congenital defects as well as acquired conditions that result from acute kidney injury (AKI) or chronic kidney disease (CKD) [3-7]. AKI involves a rapid loss of kidney function from sudden renal cell damage, which can be triggered by ischemia, toxins, or sepsis [4-6]. CKD is typified by the progressive loss of kidney function over time due to fibrosis and the erosion of healthy tissue [7]. Kidney disease leads to organ failure, known as end-stage renal disease (ESRD), which requires renal replacement therapy with dialysis or transplantation. Although renal failure can be managed clinically, it has high mortality rates and necessitates intensive, long-term care. This places a considerable burden on patients and their families, and a tremendous socioeconomic strain on healthcare systems [2].

The need for new methods to alleviate, cure, or prevent renal disease has fueled great interest in the topic of kidney stem cell biology. For over a decade now, the use of stem cells for regenerative medicine has been broadly heralded as the coming of a new age in healthcare [8]. Unfortunately, the sheer magnitude of the excitement, hope, and promise surrounding the notion of stem cell therapies has been rivaled by the enormity of the challenges in making such approaches a reality. A major hurdle facing nephrology researchers is that the human kidney has been classically defined as a non-proliferative and non-regenerative organ. However, with the discovery of adult stem cells in organs that were once thought to be non-regenerative (like the brain), the cellular make-up of the kidney has come to be reevaluated. There is emerging evidence that human kidneys possess innate regenerative abilities. For example, diabetic patients with CKD exhibited reversion of fibrotic lesions in their kidneys ten years after receiving a pancreas transplant [9,10]. Recent research efforts have focused on better understanding how the kidney can heal after injury, and determining if the kidney contains stem cells that mediate tissue replacement. Here we define the roles of stem cells in kidney development, discuss how the adult kidney responds to damage, and explore the current knowledge about the existence of adult renal stem cells.

## Review

### Stem cells and their roles in development and regeneration

**Figure 1:**
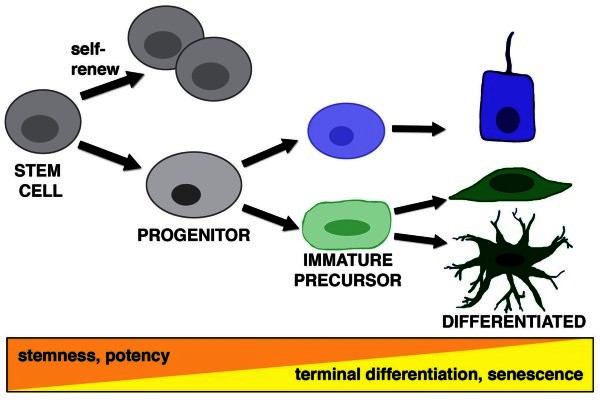
**The definition of a stem cell.** (Top) Stem cells are characterized by having the dual capacity to self-renew and produce differentiated offspring upon division. Stem cell progeny can include progenitor cells that have substantial potency to proliferate and produce one or more immature precursors that precede the differentiated state. (Below) There is a continuum of potency that becomes progressively restricted as cells move from a state of ‘stemness’ to that of terminal differentiation.

Stem cells are unique cells that are able to replenish themselves through self-renewal and can divide to produce differentiated (i.e. specialized) cell types (Figure 1) [8]. Stem cells can self-renew through a symmetric cell division, where both the newly produced offspring cells maintain the characteristics of the parent stem cell. Stem cells can also self-renew through asymmetric division, in which they produce a stem cell and another offspring that has a different potency and lineage-potential, such as a committed progenitor that transiently amplifies to make several progeny (Figure 1). Interestingly, stem cell division does not always have to involve renewal, and can instead generate assorted combinations of offspring—like two progenitors or a progenitor and a differentiated cell. The signals that trigger stem cell division and progeny identity are influenced by a complex interplay of intrinsic (cell autonomous) factors as well as extrinsic factors such as those present at the niche, which is the local microenvironment where the stem cell resides.

Stem cells drive normal development and play many roles in adult tissue regeneration [11]. Pluripotent stem cells capable of giving rise to any cell lineage can be isolated from early stage mammalian embryos. These embryonic stem (ES) cells maintain pluripotency when cultured in the right conditions [12]. As development progresses, lineage-restricted stem cells produce the tissues and organs of the body. Development does not necessarily exhaust stem cell pools, and often leads to the formation of tissue-specific or so-called adult stem cells that are maintained throughout life. Adult stem cells typically show more restricted potency (e.g. they are multi-, bi, or unipotent), and have been discovered in diverse creatures from simple metazoans like flies to mammals like mice and humans [13]. In fact, adult stem cells have been reported in a substantial list of human tissues and organs. Adult stem cells can act to replace cells that have a naturally limited lifespan, thus serving to maintain the integrity of the soma. Further, many adult stem cells can respond dynamically to injury and fuel substantial regeneration of damaged tissues. For these reasons, adult stem cells are thought to have profound impacts on the etiology of disease, malignancy, and aging [14,15]. Research efforts to identify regenerative therapeutics have encompassed the study of ES cells, adult stem cells, and the pursuit of reprogramming methods to manipulate differentiated cells and obtain induced pluripotent stem (iPS) cells that have broad lineage potential similar to the ES cell [12].

### Renal complexity: the kidney arises from stem cells during development that produce diverse differentiated cell types

The kidney develops from several stem cell pools during organogenesis. As in other mammals, the human kidney derives from intermediate mesoderm (IM) and proceeds through three progressive phases, each marked by the formation of a more advanced kidney: the pronephros, which is a rudimentary and non-functional organ, the mesonephros, which only functions a short time during embryonic development, and the metanephros, which becomes the definitive form of the adult kidney [16]. The basic structural unit of each kidney form is the nephron, an epithelial tube that accomplishes waste excretion. Nephrons have three major parts: (1) a glomerulus that filters the blood, (2) a tubule that modifies the filtrate to reabsorb and secrete solutes as the fluid passes through proximal, intermediate, and distal segments, and (3) a duct that carries the urine into a centralized collecting system [17]. The pronephros and mesonephros are made from IM that develops into simple nephrons that are connected to a pair of nephric (Wolffian) ducts, and these tissues degenerate in succession as the metanephros forms. The metanephric kidney is produced when a localized region of the nephric duct forms an outgrowth known as the ureteric bud (UB). The UB invades the adjacent IM, which at this stage is termed the metanephric mesenchyme (MM), and undergoes reiterative branching morphogenesis to create a complex duct network. The MM aggregates and condenses to form the cap mesenchyme (CM), a self-renewing stem cell population that makes nephrons around the branching UB [18-21]. Nephrogenesis proceeds when a cluster of CM undergoes a mesenchymal to epithelial transition (MET) into a renal vesicle. Each renal vesicle will proliferate and undergo morphogenesis to make one nephron. The loosely packed surrounding MM produces interstitial stroma. There is ongoing work to characterize the UB and MM derivatives, and to profile genes expressed by subsequent renal cell lineages, with a large focus on the CM because of its self-renewal property [18-21]. Although the CM exhibits self-renewal, the CM is transient and the entire CM population is triggered to undergo a final round of nephrogenesis at the end of metanephros formation. There is no evidence that a CM population is maintained to become an adult renal stem cell population. It is currently unknown if self-renewal is a short-lived property of CM renal stem cells and/or if developmental signaling induces all of the CM cells to become renal vesicles that undergo nephrogenesis.

**Figure 2:**
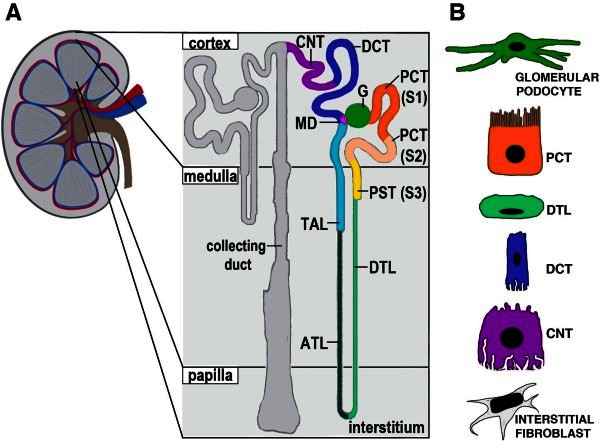
**The human kidney is comprised of heterogenous cell types.** (**A**) The kidney is comprised of a cortex, medulla, and papilla regions. Nephrons are segmented epithelial subunits of the kidney. (Right nephron) Nephron segments are color-coded to depict the regionalized structure, and show varying lengths of intermediate segments throughout the medulla and papilla region, where tracks of the collecting duct system are also found. (**B**) Examples of differentiated nephron cell types, which display unique morphological features. Abbreviations are as follows: ATL, ascending thin limb; G, glomerulus; CNT, connecting tubule; DCT, distal convoluted tubule; DTL, descending thin limb; MD, macula densa; PCT, proximal convoluted tubule; PST, proximal straight tubule; TAL, thick ascending limb.

When metanephros development is complete, this kidney has an elaborate, arborized architecture and diverse cellular makeup due to the combined contributions of CM, MM and UB progenitors. For example, over twenty differentiated cell types have been annotated in the human kidney [17] (Figure 2). Kidney composition is conserved between mammals and even with lower vertebrates like fish [17,22]. Nephrons alone contain more than a dozen epithelial cell types with specific functions (examples in Figure 2B)—which attests to the multipotency of CM stem cells during development [17]. The absolute number of nephrons and their architecture around the collecting system also renders complexity to the metanephric kidney. For example, human nephron endowment ranges from several hundred thousand to over one million nephrons per kidney, being quite variable between individuals [23,24]. Nephrons are found throughout the cortex, or outer zone of the kidney, while their tubule loops and the collecting duct system inhabit the inner zones of the medulla and papilla. Located between nephrons and the collecting system is a heterogeneous interstitial stroma inhabited by several types of fibroblasts (Figure 2B), as well as immune cells such as dendritic cells [17]. The kidney is also highly vascularized, with extensive capillary networks of endothelial cells and pericytes [17]. Finally, lymphatics are present throughout the cortex, and the kidney is innervated by fibers of the sympathetic and parasympathetic nervous systems [17].

The identification of stem cells in these adult renal structures has been riddled with controversy. Several locations in nephrons, the interstitium, and the collecting system have been cited as housing cells with proliferative potential. In some cases, there is evidence that proliferative capacity may exist in differentiated cells. Other experimental data supports the notion of ‘stemness’ in particular renal cells. In the following sections we discuss several relevant phenomena in kidney biology: first, the adolescent growth of the kidney and nephron hypertrophy, followed by sections exploring the renal response to damage and the evidence concerning the role(s) of putative kidney stem cells and differentiated cells.

### Renal cell dynamics in healthy kidneys: cell turnover and hypertrophy

During juvenile life, the mammalian kidney undergoes substantial normal growth even though organogenesis is complete just before or immediately following birth. Further, kidneys have been documented to undergo a dramatic compensatory response to the sudden removal of renal tissue.

#### *Nephrogenesis in humans is limited to early life*

**Figure 3:**
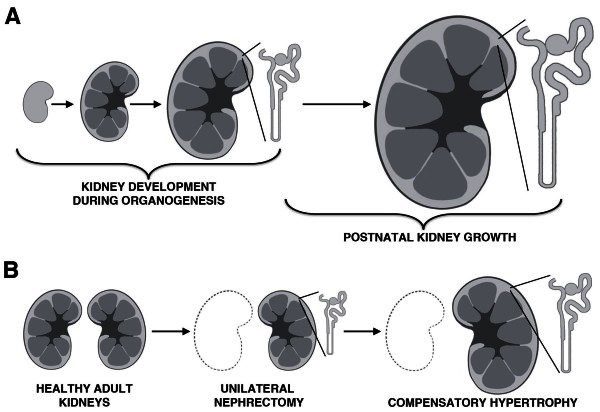
**Kidney growth and compensatory hypertrophy occurs in healthy kidneys.** (**A**) Nephrons are produced during kidney ontogeny, and subsequently grow during juvenile/adolescent life, thus exhibiting hypertrophy with age in response to changing demands on renal use and net nephron functionality throughout the kidneys. (**B**) Following unilateral nephrectomy, there is a dramatic compensatory hypertrophy response in animal models, in which the kidney expands in size due to the hypertrophy of individual nephrons.

Nephron production, or nephrogenesis, is not the stratagem for kidney maintenance in mammals. Rather, nephrogenesis is limited to gestation or early post-natal life. Human nephrogenesis ceases around week 36 of gestation [25], while nephrogenesis subsides soon after birth in rats and mice [26,27]. Although nephron endowment is set early, mammalian kidneys grow remarkably in size and functionality during juvenile stages: as body mass increases, overall kidney size and blood filtration rates increase (Figure 3A) [28]. Nephrons grow by hyperplasia and hypertrophy, leading to enlargements in glomerular size and tubule length that correlate with the elevated capacity of the kidney to filter the blood [28-30].

#### *Cell turnover and hyperplasia during juvenile kidney growth and adult homeostasis*

Tissues throughout the mammalian body exhibit varying rates of cellular turnover during adult life [11]. Epithelia that are faced with high degrees of environmental stress use virtually constant turnover rates of adult stem cells and/or their transiently amplifying progeny as a strategy to maintain tissue integrity, as seen in the skin [31] and lining of the gastrointestinal tract [32] where millions of cells are replaced daily. Kidney nephrons and collecting duct epithelia are exposed to continual passage of filtrate, and thousands of living cells from the healthy human urinary tract are excreted each day. For example, counts of exfoliated nephron tubular cells numbered ~78,000 cells per hour in men and ~68,000 cells per hour in women [33]. Cells from this so-called urinary sediment can be isolated and cultured, and include epithelial cells shed from the kidney, ureters, bladder, and urethra [33-36]. While the magnitude of renal cell turnover is lower than other organs, homeostatic mechanism(s) are still needed to maintain kidney functionality.

In adult kidneys, cell proliferation continues, albeit at a reduced rate. Renal cell division has been documented in several locales with pulse-chase labeling studies in rodents. After providing a pulse of a nucleotide analog (such as tritiated thymidine [thymidine-H^3^] or 5′-bromo-2′deoxyuridine [BrdU]), its incorporation into DNA enables the assessment of nuclear replication in preparation for mitosis. Further, the duration of time that the analog is maintained can be used to extrapolate the cycling rate of the label retaining cells (LRCs). A classic study using adult rats reported thymidine-H^3^ throughout nephron glomeruli, tubules, and the collecting system after a short chase of 8, 24, or 72 hours [37]. More recent BrdU pulse-chase studies in adult rats found LRCs in glomeruli, tubules and collecting ducts after 7 days of BrdU administration, and scattered LRCs in proximal and distal nephron tubules after a 2 week chase [38,39]. Comparisons of proximal tubule proliferation in juvenile and adult rats using BrdU pulse-chase and immunohistochemistry with mitosis markers showed that juveniles had division rates that were ~10 fold higher [40-42]. Healthy human kidneys also have dividing cells in nephron tubules based on staining for the cell division markers Ki67 and proliferating cell nuclear antigen (PCNA) [43]. Interestingly, both healthy rat and human kidneys have tubule cells positive for cyclin D1—suggesting they are in the G1 phase of the cell cycle [42]. This finding is the basis of a hypothesis that a pool of renal cells is poised for division and may serve to bastion against intermittent single cell loss or even more widespread, catastrophic insults [42].

#### *Compensatory renal hypertrophy*

Healthy mammalian kidneys can also undergo remarkable adaptive changes in response to the elimination of renal tissue. After unilateral nephrectomy, or the removal of one kidney, the remaining kidney increases in size through a process of compensatory renal hypertrophy (Figure 3B) [44,45]. The individual nephron cells in the residual kidney enlarge. For example, nephron tubular cell volume increases in proximal and distal segments [44]. The nephrons also show functional adaptations, dramatically increasing their filtration and reabsorption rates [44,45]. Thus, a kidney can respond to shifts in physiological demands posed by marked, sudden organ loss by mechanisms that include cellular proliferation and/or hypertrophy. However, the ability of the kidney to regenerate varies widely following extensive direct injuries to cells within nephrons or the interstitial environment, as discussed in the next several sections that explore regeneration phenomena in the nephron tubule and glomerulus.

### Nephron tubule epithelium can be regenerated after AKI

**Figure 4:**
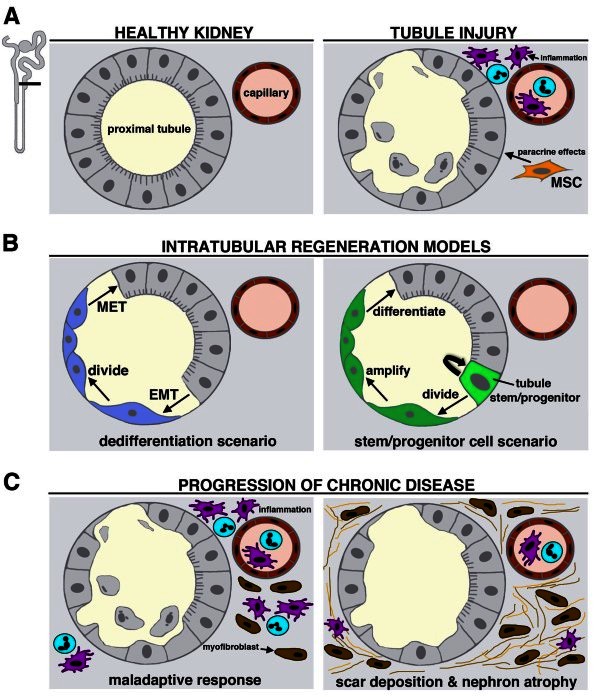
**Composition of nephron tubules in health and disease.** (**A**) (Left) Healthy tubule with an intact epithelium. (Right) After injury, tubules show the loss of epithelial cells into the luminal space, which denudes the basement membrane and is accompanied by local inflammation. Mesenchymal stem cells (MSCs) provide restorative paracrine effects to damaged tubules. (**B**) Models of intratubular regeneration in AKI include (left) the dedifferentiation of endogenous differentiated tubule epithelium and (right) the activation of renal stem/progenitor cells. (**C**) (Left) In CKD, tubular damage is not restored and this is accompanied by a prolonged inflammatory response and the activation of myofibroblasts. (Right) Over time, lesions from myofibroblasts replace once-healthy renal tissue and this fibrosis is associated with permanent nephron erosion.

Studies in animal models dating back several decades have documented that AKI causes nephron tubule cell death and local inflammation, followed by high cell proliferation that restores tubule structure and function (Figure 4A). Proximal tubule cells are particularly sensitive to AKI from toxins, as they are intensely exposed to blood-borne compounds that enter the tubule after filtration, and from ischemic insults due to their high metabolism. In fact, AKI research has largely focused on the proximal S3 segment (see Figure 2A), which shows the highest rate of death subsequent to these types of acute insults. Seminal investigations using rat kidneys documented a massive rise in the tubular mitotic index and incorporation of thymidine-H^3^ several days after mercuric chloride exposure [46]. Tubule repopulation was also observed by histology after toxic exposure to the antibiotic gentamicin [47]. Today, similar observations have been made in AKI models of ischemia reperfusion injury (IRI). Extensive proliferation in rat proximal tubules after IRI was reported based on elevated numbers of PCNA positive cells [48]. In BrdU pulse-chase studies after IRI in adult rats, nephron tubules showed increased numbers of scattered LRCs that were mostly PCNA positive [38]. Pathology studies of kidney samples from human AKI patients have also documented prominent numbers of intratubular PCNA positive cells [49], consistent with the notion that a sizeable proliferation response can occur in people after nephron damage. The explanation as to why patients differ widely in their ability to regain renal function after a bout of AKI is a resounding mystery, likely complicated by an intricate host of genetic factors, the environment, and age. Nevertheless, these studies indicate that mammalian nephron tubules (in principle) can harbor cells with a robust proliferation capacity that are sufficient in the right context to accomplish epithelial replacement.

### An intratubular cell source fuels nephron tubule epithelium regeneration after AKI

The origin of tubular dividing cells after AKI has been debated between several scenarios (Figure 4B). In the first model, surviving tubular cells dedifferentiate and undergo an epithelial to mesenchymal transition (EMT) to become a proliferating, migratory populace [4]. The tubular cells proposed to undergo an EMT switch have included those tubular cells detected in G1 in uninjured nephrons [42]. Following sufficient division, the mesenchymal cells are then proposed to differentiate into epithelium (MET) [4]. This model was based on the observations that surviving tubule cells had a flattened, squamous appearance [46,47], expressed prototypical mesenchymal markers [48], and lost polarity [50-52]. The idea of dedifferentiation was based on the expression of developmental genes by these squamous cells, including the transcription factor Paired box 2 (Pax2) [53], the neural cell adhesion molecule (NCAM) [54], and other nephrogenic genes [55]. Later, a second model was proposed in which the kidney received outside contributions from the bone marrow, which is home to multipotent hematopoietic stem cells and mesenchymal stem cells (MSCs) [56,57]. In this scenario, bone marrow cells migrated to the damaged tubules, where they engrafted and transdifferentiated to make tubule epithelium [56,57].

Subsequent lineage tracing in murine IRI models has provided compelling evidence that nephron epithelial regeneration originates from an intratubular cell source [58-60]. Chimeric mice in which the bone marrow was labeled with either a transgenic marker (e.g. LacZ, eGFP) or harbored a Y-chromosome showed no contribution of cells with these labels to regenerating tubules [58]. Reciprocally, studies that labeled tubular cells by transgenic methods showed that regenerated tubules contained only offspring with the marker and were not diluted with unmarked cells [59,60]. While these studies ruled out the scenario that bone marrow-derived cells directly contribute to nephrons, bone marrow cell contributions were confirmed in the renal interstitium [58,59]. There is now evidence that the MSCs promote nephron recovery after AKI through paracrine effects that prevent apoptosis and enhance proliferation (Figure 4A) [61-65]. This suggests that there may be a therapeutic benefit for MSC administration (see conclusions section).

### The nature of regenerating intratubular cells in AKI: differentiated, ‘progenitor’ or stem?

Further studies of nephron tubule regeneration have interrogated whether the new cells are truly the progeny of surviving epithelia that dedifferentiated, or are instead the offspring of adult renal stem cells that reside within nephrons (Figure 4B). Adult stem cells are typically rare and possess features that unequivocally distinguish them from their differentiated descendents—usually, a combination of discrete morphological and molecular traits. Historically, the existence of tubular stem cells was greeted with considerable skepticism due to the lack of any histological or expression data that suggested nephrons had unique subsets of cells among the differentiated epithelial populace. The existence of tubular stem cells has been revisited in recent studies. Researchers have reexamined the morphological features of tubule cells, reassessed the origins of tubular cells after damage, and interrogated whether subsets of tubule cells expressed genes that mark adult stem cells in other tissues.

Conflicting information about the proliferation capacities of tubular cells in animal models has been collected with label retention and lineage tracing experiments. Through long pulse-chase experiments, rare tubular LRCs were seen in cortical nephrons of 2-month old rat kidneys when BrdU was administered just three days after birth—suggesting that healthy tubules contain cells that divide infrequently [66]. Others have captured a quite different proliferation dynamic following injury. When sequential pulses of two different DNA analogs were administered after IRI in mice, epithelial cells in damaged nephrons showed co-labeling frequencies consistent with stochastic division of many tubular cells [67]. Since high numbers of tubular cells were not co-labeled, the authors concluded that the repair was unlikely to involve a tubular stem cell [67]. However, it is possible that this conclusion is difficult to extrapolate because the timing parameters of this double pulse-chase did not rule out the possibility that a subset of intratubular cells proliferated quite rapidly just after injury. Indeed, another research group reported a specific subset of proximal tubule cells in mice that expressed nuclear factor of activated T-cells cytoplasmic 1 (NFATc1) and proliferated extensively following epithelial injury from mercuric chloride exposure [68]. The discovery of the NFATc1+ proliferative tubule population is consistent with the notion that nephrons contain a particular subset of cells that divide after injury.

A recent body of evidence suggests human nephrons contain rare proliferative cells with stem cell features (Table 1) [69-73]. Stem cells express unique antigens and can be identified with antibodies that detect these markers. Cell surface antigens used for isolating renal stem cells are mostly based on known markers for other adult stem cells, and further studies are still needed to elucidate whether other markers could be used to identify renal stem cells correctly and efficiently [69-73]. The use of antigen strategies have led several groups to isolate tubule cells that exhibit impressive proliferation capacities in vitro and when administered to mice with AKI, which they termed ‘renal tubular progenitors’ [69-73].

**Table 1:** Characterization of renal progenitors from adult human kidneys

**Origin & characteristics of human renal progenitor**	***In vitro *capacity**	***In vivo *renal injury model transplantation outcome**	**Reference**
CD133+, Pax2+ from renal cortex	Self-renew, make tubular structures	Engrafted into tubules of SCID mice with glycerol-induced tubulonecrosis	[69]
CD133+, CD24+ in proximal & distal tubules	Differentiate into renal epithelium & make tubular structures	N.D.	[70]
Aldh^high^, CD133+, CD24+ from proximal tubule	N.D.	N.D.	[71]
CD133+, CD24+, CD106- in proximal & distal tubules	High proliferative capacity; differentiate into podocyte & tubular lineages	Engrafted into tubules of SCID mice with rhabdomyolysis induced AKI	[72]
CD133+, CD24+, VIM+, observed in proximal tubule	N.D.	N.D.	[73]

Discrete antigen combinations have isolated renal progenitors that show remarkable potency in both in vitro culture and transplantation settings as indicated.

Renal tubule progenitors were first reported based on a study that isolated tubule cells expressing the antigen CD133 (a marker of hematopoietic stem cells) and Pax2 from human renal cortex samples [69]. The CD133+ Pax2+ cells self-renewed and differentiated into tubule-like structures *in vitro*, and incorporated into tubules when injected into severe combined immunodeficiency (SCID) mice with glycerol-induced tubulonecrosis [69]. Another group isolated CD133+CD24+ cells from human cortical tissue and found that this fraction could expand, differentiate into renal epithelial cells, and make tubule-like structures *in vitro*[70]. The criterion of aldehyde dehydrogenase (ALDH) activity, which labels several adult stem cell types, has also been used to assess the kidney [71]. An ALDH^high^ population was isolated from proximal tubules, and shown to express CD133+CD24+ and mesenchymal markers like vimentin (VIM) [71]. A recent study reported that a human CD133+CD24+CD106- tubular progenitor population, present in both proximal and distal segments, became the predominant regenerating population in both acute and chronic tubular damage patients [72]. When isolated and injected intravenously into SCID mice with rhabdomyolysis-induced AKI, human CD133+CD24+CD106- cells engrafted into the nephrons, generating tubular cells [72]. A panel of markers that may give further insight into tubular progenitor fractions has been collected, and several unique morphological traits of CD133+CD24+VIM+ human proximal tubule cells were also noted: namely, an absence of the brush border, reduced mitochondria number and reduced cytoplasm [73]. Analogous cells were not found in healthy rat kidneys, but were detected after unilateral ureteral obstruction (UUO), a damage model for renal fibrosis [73].

More research is needed to reconcile between the conflicting data concerning how nephrons are repaired: namely whether differentiated tubular cells or renal progenitors are the source. This controversy has not been conclusively resolved. The evidence of renal tubule progenitors in human kidneys is quite compelling at present, but nevertheless remains to be substantiated in other mammalian kidneys. Additional work is needed to elucidate if the renal tubular progenitors are *bona fide* stem cells. Stem cells are operationally defined based on data indicating that they self-renew and make differentiated progeny *in vivo* during tissue homeostasis and/or injury. Lineage tracing to assess the clonogenicity of renal progenitors is needed to track their progeny in the kidney. Such studies will determine the most appropriate nomenclature for these cells. A current challenge is the need to identify specific promoters that can be used to make the necessary transgenic strains in the murine or other models. This will necessitate determining markers of these cells in animal models, though attractive candidates, such as NFATc1, already exist.

Further studies are also needed to delineate the relationship(s) and functional distinctions (if any) between renal progenitors that are positive for different antigen combinations. In addition, it will be interesting to reconcile whether or not these cells are poised in G1, or if this attribute is indicative of some other tubular fraction(s). As renal tubular progenitor subsets were found in both the proximal and distal tubule [72], it will also be interesting to determine the relationship and proliferation abilities of these cells across different nephron segments.

### Nephron tubule damage and maladaptive regeneration in the setting of CKD

Although AKI research highlights the inherent reparative ability of the nephron tubule, this attribute is unable to counteract tubular damage in the CKD fibrotic microenvironment. CKD includes a heterogenous spectrum of clinical conditions. Nevertheless, CKD pathogenesis shares the characteristic of escalating fibrosis, often initiating with a primary injury (cell loss or abnormal behavior) located at the glomerulus or tubule (Figure 4C). Fibrosis can proceed at the glomerulus (further discussed in the next section) or in the tubulointerstitial space. These lesions can propagate over many years, causing nephron dysfunction, atrophy, and collapse, coincident with damage to the vasculature that magnifies fibrogenesis and propagates a vicious damage cycle [74]. However, diabetic patients with pancreas transplants evinced amelioration of glomerular and interstitial CKD lesions after ten years [9,10]. Thus, renal scar regression was possible when patients were normoglycemic over a long period of time [9,10]. The mechanism(s) responsible for this remodeling remain unknown. Further, a recent clinical trial documented improved kidney function in ~3% of patients with hypertensive CKD over an extended time period (up to twelve years)—raising more questions about CKD prognosis [75]. Despite these reports, the most prevalent outcome for CKD patients is worsening renal function and ongoing nephron loss.

The sequence of kidney fibrosis has been likened to a wound healing process that goes terribly awry [76]. Renal cell damage is known to trigger the release of cytokines and other signals that trigger inflammation events from which myofibroblasts emerge. The origin of myofibroblasts has been quite contentious. Past candidates have included interstitial fibroblasts [76], tubular cells that undergo EMT [77-79], and endothelial cells [80,81]. Recent lineage tracing has indicated a pericyte/perivascular fibroblast origin [82].

Whether there are disease situations where myofibroblasts emerge from more than one cell compartment remains speculative. Elucidating the local cells and signals that influence myofibroblasts will be important to appreciate the cross talk that governs the progression of fibrosis in different settings. Understanding more about nephron tubular regeneration may provide viable clues as to how nephron loss can be diminished in CKD. In fact, abnormal tubular repair after AKI is thought to be one way to initiate CKD, and the severity of AKI is a robust predictor for the development of CKD [83]. There is also data from animal studies showing that tubular regeneration capacity diminishes with repeated insults: mice subjected to a single round of targeted destruction of tubular cells in the S1 and S2 proximal segments could regenerate through extensive tubular proliferation, but three rounds of targeted injury led to varying degrees of interstitial fibrosis [84]. It is unknown if this is the case in the S3 and other tubular segments. These findings suggest that work is needed to address how tubular regenerative capacity changes over time, and how it is influenced by past kidney health and the general progression of aging. It remains puzzling why some renal insults are robustly countered with successful regeneration while other injuries lead to futile or actually more damaging cellular responses. While discovering ways to enhance diminished tubule regeneration may provide a viable avenue to protect against nephron loss, the amelioration of myofibroblast activity is still likely to be a keystone for CKD treatment.

### Glomerular activities of parietal epithelial (stem?) cells: roles in growth, podocyte turnover, and maladaption in CKD

#### *The discovery of glomerular parietal epithelial cells with proliferative potential*

**Figure 5:**
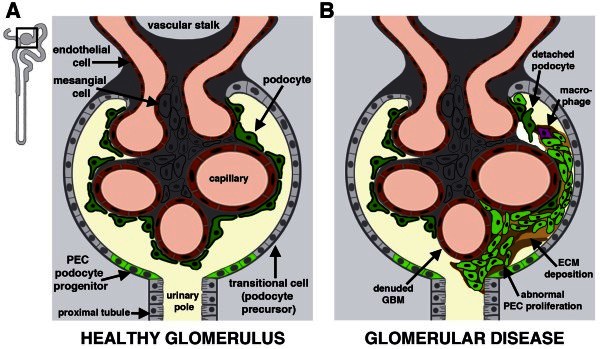
**Glomerulus homeostasis and changes in disease: the activities of PEC podocyte progenitors.** In the healthy kidney (**A**), PECs located at the urinary pole are proposed to produce podocyte precursors that migrate around Bowman’s capsule to replace differentiated podocytes lost during normal nephron function. The link between PECs and tubular progenitors has not yet been resolved, though in vitro evidence suggests that PECs may be multipotent, with the ability to generate podocytes and tubular epithelial cells. (**B**) Glomerular disease is typified by hallmark lesions in which progressive scar formation is caused by the accumulations of cells and extracellular matrix (ECM) in the Bowman’s space that can include the denuding of the glomerular basement membrane (GBM) via podocyte detachment. An advanced sclerotic lesion is depicted. The pathogenesis of glomerular disease is thought to emerge from insults to podocytes and/or the activation of abnormal PEC proliferation.

The glomerulus is comprised of at least four cell types: a perimeter of parietal epithelial cells (PECs) that form the extra-glomerular tuft, and a central glomerular tuft consisting of podocytes, endothelial cells, and mesangial cells (Figure 5A). Recent studies have suggested that some PECs produce podocytes and exhibit stem cell features in several contexts [85-88]. Distinct subsets of PECs were first reported based on differential expression of CD24 and CD133 in human kidneys [85]. PECs expressing CD24+ and CD133+ were found in close proximity to the urinary pole, which is the junction between the glomerulus and tubule [85]. When isolated and grown in culture, CD24+CD133+ PECs exhibited high colony-forming capacity and self-renewal potential [85]. They could differentiate into cells that acquired markers associated with a variety of cell types (e.g. proximal or distal tubule cells, adipocytes, osteogenic cells or neurons) when grown in appropriate conditions [85]. Further, CD24+CD133+ PECs contributed to damaged tubules and restored kidney function when injected into SCID mice with rhabdomyolysis-induced AKI [85]. Next, a CD24+CD133+ PEC population was found at the urinary pole in developing human kidneys [86]. The embryonic CD24+CD133+ PECs had similar proliferative capacities as their adult counterparts with *in vitro* culture tests [86]. Interestingly, when they were tested with an *in vivo* xenograft administration assay, they displayed higher rates of tubular engraftment possibly reflecting enhanced regenerative potential [86].

Further scrutiny of the CD24+CD133+ PEC population based on expression of the podocyte marker podocalyxin (PDX) suggested a hierarchy of lineage restriction: in culture, CD24+CD133+ PDX- PECs produced cells that expressed tubule or podocyte markers, while CD24+CD133+PDX+ PECs produced just podocytes [87]. Further, only CD24+CD133+PDX- PECs improved kidney function when administered to mice with adriamycin nephropathy, engrafting into glomerular and tubular structures [87]. Interestingly, a discrete spatial arrangement of these cells was noted in the glomerulus, with CD24+CD133+PDX- PECs at the urinary pole, CD24-CD133-PDX+ podocytes at the vascular pole, and another epithelial population situated in between that were CD24+CD133+PDX+ [87].

#### *The new working model of podocyte ‘stem’ cells*

These findings led to the proposal of a largely compelling model that PECs located at the urinary pole are in fact podocyte stem cells that upon division produce progenitors that migrate slowly around the capsule and eventually replace lost or damaged podocytes [87]. It is known that humans shed podocytes into the urine when the kidney is healthy, and patients with glomerular diseases excrete elevated numbers of podocytes (up to 400 fold more) [35]. Podocytes from the mouse and rat can undergo hypertrophy in culture in response to mechanical stress [88,89] and high glucose media [90], and human podocytes undergo hypertrophy in disease states such as diabetes [91,92]. However, hypertrophy is not thought to account for podocyte shedding in healthy kidneys, and podocytes themselves are post-mitotic based on pulse-chase studies [93]. Thus, the PEC stem cell model proffers an explanation that accounts for podocyte homeostasis over life. In support of the PEC stem cell model, genetic lineage data from mice tracked the ability of PECs located at the glomerular urinary pole to differentiate into podocytes during and after post-natal nephrogenesis [94]. These findings implicate PECs as the means for normal glomerular growth, and are consistent with the idea that PECs serve to maintain podocyte numbers. Additional lineage tracing in adults is needed to further support the model. Other studies that isolated self-renewing cell lines from human and mouse glomeruli reported that these cells express various developmental genes [95,96]. Uncovering the roles of these genes could provide useful mechanistic insights into PEC behavior.

There is experimental evidence that abnormal PEC proliferation after glomerular injury can lead to maladaptive glomerular lesions that elicit CKD [97,98] (Figure 5B). Pathologic changes can include hyperplastic lesions like glomerular crescents, which are accumulations of proliferating cells in the extracapillary space [97]. Over time, crescents can obstruct glomerular outflow to the tubule, leading to tubule degeneration and consequent nephron destruction. In addition, lineage tracing has shown that activated PECs can participate in sclerotic lesions in which cells invade the capsule and deposit extracellular matrix leading to progressive glomerular scarring [98], which destroys the glomerulus and causes proteinuria that ultimately damages the nephron. Further work to discover the signals leading to abnormal PEC proliferation will be crucial for understanding and obviating maladaptive PEC behavior. Toward this end, some factors that influence PEC cell fate decisions during development have been identified, such as Wnt and BMP [99], and the modulation of the Notch pathway has been implicated in balancing glomerular cell proliferation and regeneration after injury [100].

The precedence that glomerular lesions can regress comes from biopsy analysis of diabetic patients who received pancreas transplants [9,10]. Clinical studies have shown that administration of angiotensin-converting-enzyme (ACE) inhibitor reversed proteinuria and CKD progression in patients with non-diabetic chronic nephropathy [101,102] and in diabetics [103]. The mechanism for how ACE inhibition treatment triggers regression in humans is hard to address due to the difficulty in obtaining repeat biopsies. However, work in animal models showed that ACE inhibition correlated with structural changes in the glomerulus including podocyte repopulation [104] and reduced proliferation in sclerotic lesions [105]. These findings provide hope that it may be feasible to reverse, halt, or slow glomerular scarring. In turn, lessened or obviated glomerular scarring may prevent nephron atrophy.

### The renal papilla as a putative site for renal stem cells

The renal papilla, or inner medulla, is a central region of the adult kidney that contains epithelial tracks of collecting ducts and nephron tubule intermediate segments (the loops of Henle) that concentrate urine (Figure 2). Whether the papilla serves as a niche for renal stem cells has been highly controversial. Evidence that the papilla may harbor renal stem cells originated from BrdU pulse-chase experiments: LRCs were found in papilla tubules and interstitial cells, and their number was reduced following ischemia, leading to the hypothesis that the LRCs might be involved in renal repair [66]. Plasticity of the papilla cells was tested *in vitro*, where some papilla cells proliferated to form spherical cell aggregates that co-expressed epithelial and mesenchymal markers [66]. The proliferation and migration of LRCs was also observed during normal homeostasis, and their number decreased with age, suggesting they may represent a renal stem cell pool responding to IRI [106]. Conflicting labeling results were also reported—namely that the papilla LRCs became quiescent early during renal organogenesis [107].

Meanwhile, the renal papilla was proposed to be a stem cell niche when marker-based approaches were employed. Nestin is expressed in multi-lineage stem cells in brain and mesonephric mesenchyme [108]. By examining Nestin-GFP expression in transgenic mice, a large number of Nestin-expressing cells were detected as clusters in the renal papilla [108]. Nestin expression has been correlated with CD133, and Nestin+CD133+ cells were found extensively in the renal papilla [109]. The plasticity of these cells was tested through *in vitro* culture assays, where the cells showed the capacity for tubulogenesis [109].

Despite this body of evidence, not all approaches have supported the notion that the papillary region harbors renal stem cells that migrate to injured regions of the kidney. For example, the migration and proliferation of papillary LRCs after renal IRI was refuted by other BrdU pulse-chase experiments that failed to observe similar events [67]. In another report, the expression of telomerase reverse transcriptase (mTert) expression was surveyed after kidney injury because this gene marks embryonic and adult stem cells [110]. Expression of mTert could be detected in a subset of papillary epithelial cells, but this population neither divided nor migrated out of the renal papilla during repairing phases, suggesting they were not a progenitor-cell population [110]. While the existence of stem cells in the papilla remains an area of research filled with contradictory reports, it is reasonable to speculate that there must be some molecular mechanism(s) to support cell turnover and damage in this kidney region.

### The problem of resolving disparate reports of putative renal stem cell populations

Many laboratories have reported finding kidney cells with stem cell characteristics that may correspond to one, several or even none of the cell types already discussed. Therefore, a significant and unresolved issue is whether the cells described by different researchers are distinct or identical to each other. In the following subsections we provide a short discussion of the information gathered about various putative renal stem cells based on gene expression data and/or assays of proliferative potential.

#### *The establishment of renal stem cell lines from adult kidneys*

*In vitro* studies have been performed with LRCs isolated from adult ischemic rat kidneys after BrdU labeling, by virtue of their low Hoechst staining in the presence of BrdU [39]. In this damage context, LRCs are enriched in proximal tubules. The Hoechst^low^/BrdU+ LRCs proliferated in culture, forming tubule-like structures in conditions that enabled tubulogenesis, and incorporating into nephrons and interstitium when cultured with developing rat metanephric kidneys [39]. After isolating single cells from a healthy rat nephron, another group established a cell line that could expand greatly in culture and generate differentiated tubule cells in different culture conditions; this cell line could also engraft into rat kidney tubules after IRI [111]. However, due to the complex cellular composition of the nephron, the origin of this cell line is unclear. Another caveat in interpreting both of these studies is that the cells of origin are likely from the nephron, but could nonetheless be from other locales as well, such as the interstitium.

A multipotent population of nontubular cells was isolated from the mouse kidney based on the expression of stem cell antigen-1 (Sca-1) and the absence of lineage (Lin) markers of bone-marrow-derived stem cells [112]. Global gene expression analysis found enriched expression of mesodermal lineage genes, and that the Sca-1+ Lin- population could potentially contribute to renal repair after IRI [112]. Multipotent renal progenitor cells (MRPCs) isolated by culturing rat kidney cells showed high self-renewal capacity and expression of genes involved in kidney development, like Pax2 [113]. MRPC potency was demonstrated *in vitro*, as the cells were able to differentiate into myogenic, adipogenic, neural or osteogenic lineages; further, MRPCs differentiated into renal tubular cells after they were injected into the parenchyma of damaged murine kidneys [113]. Another group isolated what they termed mouse kidney progenitor cells (MKPC) from the interstitium of the medulla and papilla that exhibited self-renewal capacity and expressed early nephrogenesis genes [114]. Injection of MKPC rescued renal damage when administered to IRI damaged murine kidneys, and incorporation of MKPCs into some renal tubules was observed [114].

Interestingly, MSCs have been cultured from the renal capsule, which is a thin sheet of connective tissue that envelops the kidney and contains fibroblasts, adipocytes, and blood vessels [115]. After IRI, capsule cells migrated into the renal parenchyma, where they are postulated to participate in the injury response [115]. There have been other reports of MSCs found in adult kidneys, such as the isolation of MSCs able to differentiate into endothelial and smooth muscle cells that could promote vasculogenesis in IRI [116,117]. A limitation in interpreting all of these renal stem cell line studies is that culture conditions can have a significant impact on cellular behaviors that makes understanding the identity and potency of the cells somewhat convoluted in the absence of data to irrefutably track them *in vivo* during health or injury.

#### *Kidney side population studies*

Stem cells can be isolated through flow cytometry due to their ability to efflux Hoechst 33342 dye and are characterized as a so-called side population (SP). Several SP cells have been identified in various organs, such as heart, lung, and skeletal muscle. Kidney SP cells from the mouse [118,119] and rat [120] have been characterized, and expression of particular genes like Musculin/MyoR has been annotated [119]. Another group isolated a renal SP population located in the proximal tubule, supporting a ‘tubular niche’ hypothesis [121]. These SP cells were highly heterogeneous, although they showed a humoral role during renal repair without tubular integration [121]. The existence of SP cells in adult human kidneys has also been reported [122,123]. However, *in vitro* characterization demonstrated the heterogeneity of human renal SP population as well, indicating SP cells cannot be equally considered as stem cells [122]. The ability of SP cells to improve renal regeneration has been reported, and the mechanism behind this ameliorative role may be due to their generation of bone morphogenic protein 7 (BMP7), which reversed chronic renal injury when given exogenously and could be induced by treatment with a histone deacetylase inhibitor [124]. Taken together, whether kidney SP cells represent a particular stem cell population still remains controversial because renal SP cells show considerable heterogeneity. To date, the SP assay has not served to unequivocally identify kidney stem cells, and future work is needed to assess if podocyte or tubular progenitors exhibit the SP phenotype, or can be enriched using this parameter.

### Prospects for future research

It is clear that there are many gaps in our understanding about tubular, glomerular, and other speculated stem cell fractions in the kidney. However, there are several promising research venues that may provide novel insights into how regenerative kidney cells work. One such area is the study of renal development. Developmental studies are applicable for understanding regeneration because information about how kidney lineages are formed can provide gene expression data that could help pinpoint adult renal stem cells and the signaling pathways that modulate the mobilization and behavior of reparative cells. Further, knowledge from studying embryonic progenitors during kidney development may be directly relevant to adult cell types, as multipotent renal progenitors isolated from adult kidneys could represent a subset of embryonic progenitors that persist from early nephrogenesis.

Another promising topic concerns how kidney cells sense injury and interact with their environment. Understanding how kidney cells perceive and react to local damage may provide critical information about the early events in kidney disease. A fundamental player is likely to be the primary cilium [125]. Cilia are organelles that play crucial roles in how cells interact with their environment. Two types of cilia have been documented in vertebrates, motile cilia and the non-motile primary cilium [126]. Primary cilia are chemical and mechanosensors between the cell and its milieu [126]. In healthy adult human kidneys, the epithelial cells lining the lumen of nephrons and collecting ducts have primary cilia with a 9+0 microtubule structure that interact with urinary flow [127]. Cilia defects cause cystic kidney diseases, characterized by abnormalities in nephron tubular epithelial cells that lead to their overproliferation and development of fluid-filled cysts. Since changes in urinary flow during AKI and CKD are likely sensed by tubule residents through the primary cilium, this signal transduction may have important ramifications for how tubular cells recognize injury. Thus, primary cilium signaling may hold a prominent position in the course of events that lead to nephron regeneration or maladaptive responses.

## Conclusions

New therapeutic options are needed to mitigate kidney disease. The intricate cellular composition of the kidney poses challenges both to understanding and treating kidney disease. The presence of many renal cell types likely means that the microenvironments in the kidney are manifold and diverse. Nevertheless, the discovery of regenerative renal cells in adult kidneys—especially those in the nephron tubule and PECs in the glomerulus—provides hope that there are already cell templates and conducive environs where kidney integrity can be restored by promoting regenerative mechanisms. Improvements in the detection of specific kidney diseases with diagnostic biomarkers that can reliably predict tissue alterations are crucial in the years ahead. Emerging evidence supports the notion that renal progenitors are a conserved evolutionary phenomenon, and novel future insights are likely to be gleaned from the analysis of renal progenitors across the animal kingdom [128].

One important element of creating regenerative therapeutics will involve the discovery of how to deliver and control such interventions in time and space, as cautioned by the ability of unchecked PEC proliferation to initiate glomerular lesions. Phenotypic improvements triggered by alterations in the microenvironment, as exemplified by the paracrine effects of MSCs when they lodge in renal parenchyma, may be a viable place to start. Many clinical trials with stem cell therapies are currently underway, both with MSCs and other cell types [129]. There have been encouraging outcomes with the safety of using autologous transplants, which provides preliminary reassurance that the efficacy of administering mobilized MSCs (or other cells) to ameliorate human kidney disease may be testable in the near future. Further, in experimental models of CKD, various studies have shown that BMP7 can inhibit or even reverse fibrosis, which raises the possibility that targeting the BMP7 pathway may provide one avenue to help patients with CKD [130].

Considerations of the appropriate target patient population will also need to weigh more than the kidney disease diagnosis: variables like age and the burden of co-mordibities will also be factors that come into play. Like other body organs, the aging kidney shows dramatic alterations [131]. For example, it has been appreciated for decades that proliferative capacity of the mammalian kidney declines with age [132]. However, recent experimental studies have documented that young bone marrow cells from mice could alleviate aspects of renal aging when administered to old mice [133]. This suggests that therapeutic options may be enhanced as parameters like cellular age are considered and explored.

Taken together, recent research has uncovered enticing data that several cell types within the kidney have innate regenerative capacity. It will be essential to understand how to enhance or induce regeneration in a controlled fashion that can prevent maladaptive outcomes arising from perturbed cell behavior. Although this may turn out to be a complicated line to walk, the existence of several stem cells in the kidney provides encouragement that regenerative approaches might be harnessed to combat renal disease in the not-so-distant future.

## Abbreviations

ACE: Angiotensin-converting-enzyme; AKI: Acute kidney injury; ALDH: Aldehyde dehydrogenase; BMP7: Bone morphogenic protein 7; BrdU: 5’-bromo-2’deoxyuridine; CKD: Chronic kidney disease; CM: Cap mesenchyme; ECM: Extracellular matrix; EMT: Epithelial to mesenchymal transition; ES cell: Embryonic stem cell; ESRD: End-stage renal disease; GBM: Glomerular basement membrane; IM: Intermediate mesoderm; iPS cell: Induced pluripotent stem cell; IRI: Ischemia reperfusion injury; Lin: Lineage; LRC: Label retaining cell; MET: Mesenchymal to epithelial transition; MKPC: Mouse kidney progenitor cell; MM: Metanephric mesenchyme; MRPC: Multipotent renal progenitor cell; MSC: Mesenchymal stem cell; NCAM: Neural cell adhesion molecule; NFATc1: Nuclear factor of activated T-cells cytoplasmic 1; Pax2: Paired box 2; PCNA: Proliferating cell nuclear antigen; PDX: Podocalyxin; PEC: Parietal epithelial cell; Sca-1: Stem cell antigen-1; SCID: Severe combined immunodeficiency; SP: Side population; mTERT: Telomerase reverse transcriptase; UB: Ureteric bud; UUO: Unilateral ureteral obstruction; VIM: Vimentin.

## Competing interest

The authors declare that they have no commercial or other competing interests to disclose.

## References

[B1] United States Renal Data System 2012 Annual Data Report 2012 Atlas of Chronic Kidney Disease and End-Stage Renal Disease in the United States http://www.usrds.org/adr.aspx

[B2] Weiner DE Public health consequences of chronic kidney disease Clin Pharmacol Ther 2009 2 566 569 10.1038/clpt.2009.137 19641489 PMC2788514

[B3] Schedl A Renal abnormalities and their developmental origin Nat Rev Genet 2007 2 791 802 17878895 10.1038/nrg2205

[B4] Bonventre JV Yang L Cellular pathophysiology of ischemic acute kidney injury J Clin Invest 2011 2 4210 4221 10.1172/JCI45161 22045571 PMC3204829

[B5] Murugan R Kellum JA Acute kidney injury: what’s the prognosis? Nat Rev Nephrol 2011 2 209 217 10.1038/nrneph.2011.13 21343898 PMC3547642

[B6] Venkatachalam MA Griffin KA Lan R Geng H Saikumar P Bidani AK Acute kidney injury: a springboard for progression in chronic kidney disease Am J Physiol Renal Physiol 2010 2 F178 F1094 10.1152/ajprenal.00017.2010 PMC2867413 20200097

[B7] El Nahas AM Bello AK Chronic kidney disease: the global challenge Lancet 2005 2 331 340 15664230 10.1016/S0140-6736(05)17789-7

[B8] Stocum DL Stem cells in regenerative biology and medicine Wound Rep Reg 2001 2 429 442 10.1046/j.1524-475x.2001.00429.x 11896986

[B9] Fioretto P Steffes MW Sutherland DER Goetz FC Mauer M Reversal of lesions of diabetic nephropathy after pancreas transplantation N Engl J Med 1998 2 69 75 10.1056/NEJM199807093390202 9654536

[B10] Fioretto P Sutherland DER Najafian B Mauer M Remodeling of renal interstitial and tubular lesions in pancreas transplant recipients Kidney Int 2006 2 907 910 10.1038/sj.ki.5000153 16518350

[B11] Weissman IL Stem cells: units of development, units of regeneration, and units in evolution Cell 2000 2 157 168 10.1016/S0092-8674(00)81692-X 10647940

[B12] Yamanaka S Blau HM Nuclear reprogramming to a pluripotent state by three approaches Nature 2010 2 704 712 10.1038/nature09229 20535199 PMC2901154

[B13] Hsu YC Fuchs E A family business: stem cell progeny join the niche to regulate homeostasis Nat Rev Mol Cell Bio 2012 2 103 114 10.1038/nrm3272 22266760 PMC3280338

[B14] Burness ML Sipkins DA The stem cell niche in health and malignancy Semin Cancer Biol 2010 2 107 115 10.1016/j.semcancer.2010.05.006 20510363

[B15] Sharpless NE DePinho RA How stem cells age and why this makes us grow old Nat Rev Mol Cell Biol 2007 2 703 713 10.1038/nrm2241 17717515

[B16] McCampbell KK Wingert RA Renal stem cells: fact or science fiction? Biochem J 2012 2 153 168 10.1042/BJ20120176 22574774 PMC3350370

[B17] Reilly RF Bulger RE Kriz W In Diseases of the Kidney and Urinary Tract, Eighth Edition Structural-functional relationships in the kidney 2007 Philadelphia: Lippincott Williams & Wilkins: Edited by Schrier RW 2 53

[B18] Little MH Brennan J Georgas K Davies JA Davidson DR Baldock RA A high-resolution anatomical ontology of the developing murine genitourinary tract Gene Expr Patterns 2007 2 680 699 10.1016/j.modgep.2007.03.002 17452023 PMC2117077

[B19] Brunskill EW Aronow BJ Georgas K Rumballe B Valerius MT Aronow J Atlas of gene expression in the developing kidney at microanatomic resolution Dev Cell 2008 2 781 791 10.1016/j.devcel.2008.09.007 19000842 PMC2653061

[B20] Mugford JW Yu J Kobayashi A McMahon AP High-resolution gene expression analysis of the developing mouse kidney defines novel cellular compartments within the nephron progenitor population Dev Biol 2009 2 312 323 10.1016/j.ydbio.2009.06.043 19591821 PMC2748313

[B21] Yu J Valerius MT Duah M Staser K Hansard JK Guo JJ Identification of molecular compartments and genetic circuitry in the developing mammalian kidney Development 2012 2 1863 1873 10.1242/dev.074005 22510988 PMC3328182

[B22] Wingert RA Davidson AJ The zebrafish pronephros: a model to study nephron segmentation Kidney Int 2008 2 1120 1127 10.1038/ki.2008.37 18322540

[B23] Nyengaard JR Bendtsen TF Glomerular number and size in relation to age, kidney weight, and body surface in normal man Anat Rec 1992 2 194 201 10.1002/ar.1092320205 1546799

[B24] Hughson M Farris III AB, Douglas-Denton R, Yoy WE, Bertram JF: Glomerular number and size in autopsy kidneys: the relationship to birth weight Kidney Int 2003 2 2113 2122 10.1046/j.1523-1755.2003.00018.x 12753298

[B25] Potter EL Thierstein ST Glomerular development in the kidney as an index of fetal maturity J Pediatr 1943 2 695 706 10.1016/S0022-3476(43)80226-2

[B26] Hartman HA Lai HL Patterson LT Cessation of renal morphogenesis in mice Dev Biol 2007 2 379 387 17826763 10.1016/j.ydbio.2007.08.021 PMC2075093

[B27] Solomon S Developmental changes in nephron number, proximal tubule length and superficial nephron glomerular filtration rate of rats J Physiol 1977 2 573 589 592203 10.1113/jphysiol.1977.sp012061 PMC1353643

[B28] Wesson LG Compensatory growth and other growth responses of the kidney Nephron 1989 2 149 184 10.1159/000185282 2644570

[B29] de Rouffignac C Monnens L Functional and morphologic maturation of superficial and juxtamedullary nephrons in the rat J Physiol 1976 2 119 129 994033 10.1113/jphysiol.1976.sp011588 PMC1307633

[B30] Spitzer A Brandis M Functional and morphologic maturation of the superficial nephrons J Clin Invest 1974 2 279 287 10.1172/JCI107548 4808641 PMC301463

[B31] Blanpain C Fuchs E Epidermal homeostasis: a balancing act of stem cells in the skin Nat Rev Mol Cell Bio 2009 2 207 217 19209183 10.1038/nrm2636 PMC2760218

[B32] van der Flier LG Clevers H Stem cells, self-renewal, and differentiation in the intestinal epithelium Annu Rev Physiol 2009 2 241 260 10.1146/annurev.physiol.010908.163145 18808327

[B33] Prescott LF The normal urinary excretion rates of renal tubular cells, leucocytes and red blood cells Clin Sci 1966 2 425 435 5927694

[B34] Dörrenhaus A Müller JIF Golka K Jedrusik P Schulze H Föllmann W Cultures of exfoliated epithelial cells from different locations of the human urinary tract and the renal tubular system Arch Toxicol 2000 2 618 626 10.1007/s002040000173 11201669

[B35] Vogelmann SU Nelson WJ Myers BD Lemley KV Urinary excretion of viable podocytes in health and renal disease Am J Physiol Renal Physiol 2003 2 F40 F48 12631553 10.1152/ajprenal.00404.2002 PMC3368602

[B36] Rahmoune H Thompson PW Ward JM Smith CD Hong G Brown J Glucose transporters in human renal proximal tubular cells isolated from the urine in patients with non-insulin-dependent diabetes Diabetes 2005 2 3427 3434 10.2337/diabetes.54.12.3427 16306358

[B37] Messier B Leblond CP Cell proliferation and migration as revealed by radioautography after injection of thymidine-H3 into male rats and mice Amer J Anat 1960 2 247 285 10.1002/aja.1001060305 13769795

[B38] Maeshima A Yamashita S Nojima Y Identification of renal progenitor-like tubular cells that participate in the regeneration processes of the kidney J Am Soc Nephrol 2003 2 3138 3146 10.1097/01.ASN.0000098685.43700.28 14638912

[B39] Maeshima A Sakurai H Nigam SK Adult kidney tubular cell population showing phenotypic plasticity, tubulogenic capacity, and integration capability into developing kidney J Am Soc Nephrol 2006 2 188 198 16338966 10.1681/ASN.2005040370

[B40] Vogetseder A Karadeniz A Kaissling B Le Hir M Tubular cell proliferation in the healthy rat kidney Histochem Cell Biol 2005 2 97 104 10.1007/s00418-005-0023-y 16133123

[B41] Vogetseder A Palan T Bacic D Kaissling B Le Hir M Proximal tubular epithelial cells are generated by division of differentiated cells in the healthy kidney Am J Physiol Cell Physiol 2007 2 C807 C813 16987990 10.1152/ajpcell.00301.2006

[B42] Vogetseder A Picard N Gaspert A Walch M Kaissling B Le Hir M Proliferation capacity of the renal proximal tubule involves the bulk of differentiated epithelial cells Am J Physiol Cell Physiol 2008 2 C22 C28 10.1152/ajpcell.00227.2007 17913845

[B43] Nadasdy T Laszik Z Blick KE Johnson LD Silva FG Proliferative activity of intrinsic cell populations in the normal human kidney J Am Soc Nephrol 1994 2 2032 2039 7919156 10.1681/ASN.V4122032

[B44] Hayslett JP Kashgarian M Epstein FH Functional correlates of compensatory renal hypertrophy J Clin Invest 1968 2 774 781 10.1172/JCI105772 5641618 PMC297228

[B45] Hostetter TH Progression of renal disease and renal hypertrophy Annu Rev Physiol 1995 2 263 278 10.1146/annurev.ph.57.030195.001403 7778868

[B46] Cuppage FE Tate A Repair of the nephron following injury with mercuric chloride Am J Pathol 1967 2 405 429 4227041 PMC1965336

[B47] Houghton DC Hartnett M Campbell-Boswell M Porter G Bennett W A light and electron microscopic analysis of gentamicin nephrotoxicity in rats Am J Pathol 1976 2 589 612 1258978 PMC2032419

[B48] Witzgall R Brown D Schwarz C Bonventre JV Localization of proliferating cell nuclear antigen, vimentin, c-Fos and clusterin in the postischemic kidney. Evidence for a heterogenous genetic response among nephron segments, and a large pool of mitotically active and dedifferentiated cells J Clin Invest 1994 2 2175 2188 10.1172/JCI117214 7910173 PMC294357

[B49] Nadasdy T Laszik Z Blick KE Johnson DL Burst-Singer K Nast C Human acute tubular necrosis: a lectin and immunohistochemical study Hum Pathol 1995 2 230 239 10.1016/0046-8177(95)90042-X 7860054

[B50] Molitoris BA Wilson PD Schrier RW Simon FR Ischemia induces partial loss of surface membrane polarity and accumulation of putative calcium ionophores J Clin Invest 1985 2 2097 2105 10.1172/JCI112214 3001141 PMC424317

[B51] Molitoris BA Hoilien CA Ahnen DJ Wilson PD Kim J Characterization of ischemia-induced loss of epithelial polarity J Membr Biol 1988 2 233 242 10.1007/BF01872161 2468776

[B52] Zuk A Bonventre JV Brown D Matlin KS Polarity, integrin, and extracellular matrix dynamics in the postischemic rat kidney Am J Physiol 1998 2 C711 C731 9730955 10.1152/ajpcell.1998.275.3.C711

[B53] Imgrund M Gröne E Gröne HJ Kretzler M Holzman L Schlöndorff D Re-expression of the developmental gene Pax-2 during experimental acute tubular necrosis in mice Kidney Int 1999 2 1423 1431 10.1046/j.1523-1755.1999.00663.x 10504494

[B54] Abbate M Brown D Bonventre JV Expression of NCAM recapitulates tubulogenic development in kidneys recovering from acute ischemia Am J Physiol 1999 2 F454 F463 10484529 10.1152/ajprenal.1999.277.3.F454

[B55] Villanueva S Cespedes C Vio CP Ischemic acute renal failure induces the expression of a wide range of nephrogenic proteins Am J Physiol Regul Integr Comp Physiol 2006 2 R861 870 16284088 10.1152/ajpregu.00384.2005

[B56] Lin F Cordes K Li L Hood L Couser WG Shankland SJ Hematopoietic stem cells contribute to the regeneration of renal tubules after renal ischemia-reperfusion injury in mice J Am Soc Nephrol 2003 2 1188 1199 10.1097/01.ASN.0000061595.28546.A0 12707389

[B57] Kale S Karihaloo A Clark PR Kashgarian M Krause DS Cantley LG Bone marrow stem cells contribute to repair of the ischemically injured renal tubule J Clin Invest 2003 2 42 49 12824456 10.1172/JCI17856 PMC162291

[B58] Duffield JS Park KM Hsiao LL Kelley VR Scadden DT Ichimura T Restoration of tubular epithelial cells during repair of the postischemic kidney occurs independently of bone marrow-derived stem cells J Clin Invest 2005 2 1743 1755 10.1172/JCI22593 16007251 PMC1159124

[B59] Lin F Moran A Igarashi P Intrarenal cells, not bone marrow-derived cells, are the major source for regeneration in the postischemic kidney J Clin Invest 2005 2 1756 1764 10.1172/JCI23015 16007252 PMC1159127

[B60] Humphreys BD Valerius MT Kobayashi A Mugford JW Soeung S Duffield JS Intrinsic epithelial cells repair the kidney after injury Cell Stem Cell 2008 2 284 291 10.1016/j.stem.2008.01.014 18371453

[B61] Morigi M Imberti B Zoja C Corna D Tomasoni S Abbate M Mesenchymal stem cells are renotropic, helping to repair the kidney and improve function in acute renal failure J Am Soc Nephrol 2004 2 1794 1804 10.1097/01.ASN.0000128974.07460.34 15213267

[B62] Herrera MB Bussolati B Bruno S Fonsato V Romanazzi GM Camussi G Mesenchymal stem cells contribute to the renal repair of acute tubular epithelial injury Int J Mol Med 1994 2 1035 1041 15547670

[B63] Tögel F Hu Z Weiss K Isaac J Lange C Westenfelder C Administered mesenchymal stem cells protect against ischemic acute renal failure through differentiation-independent mechanisms Am J Physiol Renal Physiol 2005 2 F31 F42 10.1152/ajprenal.00007.2005 15713913

[B64] Lange C Tögel F Ittrich H Clayton F Nolte-Ernsting C Zander AR Administered mesenchymal stem cells enhance recovery from ischemia/reperfusion-induced acute renal failure in rats Kidney Int 2005 2 1613 1617 10.1111/j.1523-1755.2005.00573.x 16164638

[B65] Bi B Schmitt E Israilova M Nishio H Cantley LG Stromal cells protect against acute tubular injury via an endocrine effect J Am Soc Nephrol 2007 2 2486 2496 10.1681/ASN.2007020140 17656474

[B66] Oliver JA Maarouf O Cheema FH Martens TP Al-Awqati Q The renal papilla is a niche for adult kidney stem cells J Clin Invest 2004 2 795 804 15372103 10.1172/JCI20921 PMC516259

[B67] Humphreys BD Czerniak S DiRocco DP Hasnain W Cheema R Bonventre JV Repair of injured proximal tubule does not involve specialized progenitors Proc Natl Acad Sci, USA 2011 2 9226 9231 10.1073/pnas.1100629108 21576461 PMC3107336

[B68] Langworthy M Zhou B de Caestecker M Moeckel G Baldwin HS NFATc1 identifies a population of proximal tubule cell progenitors J Am Soc Nephrol 2009 2 311 321 10.1681/ASN.2008010094 19118153 PMC2637056

[B69] Bussolati B Bruno S Grange C Buttiglieri S Deregibus MC Cantino D Isolation of renal progenitor cells from adult human kidney Am J Pathol 2005 2 545 555 10.1016/S0002-9440(10)62276-6 15681837 PMC1602314

[B70] Sallustio F De Benedictis L Castellano G Zaza G Loverre A Constantino V TLR2 plays a role in the activation of human resident renal stem/progenitor cells FASEB J 2010 2 514 525 10.1096/fj.09-136481 19843711

[B71] Lindgren D Boström AK Nilsson K Hansson, Sjölund J, Möller C, et al.: Isolation and characterization of progenitor-like cells from human renal proximal tubules Am J Pathol 2011 2 828 837 10.1016/j.ajpath.2010.10.026 21281815 PMC3070548

[B72] Angelotti ML Ronconi E Ballerini L Peired A Mazzinghi B Sagrinati C Characterization of renal progenitors committed toward tubular lineage and their regenerative potential in renal tubular injury Stem Cells 2012 2 1714 1725 10.1002/stem.1130 22628275

[B73] Smeets B Boor P Jijkman H Sharma SV Jirak P Mooren F Proximal tubular cells contain a phenotypically distinct, scattered cell population involved in tubular regeneration J Pathol 2013 2 645 659 10.1002/path.4125 23124355 PMC3951144

[B74] Liu Y Cellular and molecular mechanisms of renal fibrosis Nat Rev Nephrol 2011 2 684 696 10.1038/nrneph.2011.149 22009250 PMC4520424

[B75] Hu B Gadegbeku C Lipkowitz MS Rostand S Lewis J Wright JT Kidney function can improve in patients with hypertensive CKD J Am Soc Nephrol 2012 2 706 713 10.1681/ASN.2011050456 22402803 PMC3312500

[B76] Hirschberg R Wound healing in the kidney: complex interactions in renal interstitial fibrogenesis J Am Soc Nephrol 2005 2 9 11 15574504 10.1681/ASN.2004110901

[B77] Yang J Liu Y Dissection of key events in tubular epithelial to myofibroblast transition and its implications in renal interstitial fibrosis Am J Pathol 2001 2 1465 1475 10.1016/S0002-9440(10)62533-3 11583974 PMC1850509

[B78] Iwano M Plieth D Danoff TM Xiu C Okada H Neilson EG Evidence that fibroblasts derive from epithelium during tissue fibrosis J Clin Invest 2002 2 341 350 12163453 10.1172/JCI15518 PMC151091

[B79] Yamashita S Maeshima A Nojima Y Involvement of renal progenitor tubular cells in epithelial-to-mesenchymal transition in fibrotic rat kidneys J Am Soc Nephrol 2005 2 2044 2051 10.1681/ASN.2004080681 15888566

[B80] Zeisberg EM Potenta SE Sugimoto H Zeisberg M Kalluri R Fibroblasts in kidney fibrosis emerge via endothelial-to-mesenchymal transition J Am Soc Nephrol 2008 2 2282 2287 10.1681/ASN.2008050513 18987304 PMC2588112

[B81] Li J Qu X Bertram JF Endothelial-myofibroblast transition contributes to the early development of diabetic renal interstitial fibrosis in streptozotocin-induced diabetic mice Am J Pathol 2009 2 1380 1388 10.2353/ajpath.2009.090096 19729486 PMC2751535

[B82] Humphreys BD Lin SL Kobayashi A Hudson TE Nowlin BT Bonventre JV Fate tracing reveals the pericyte and not epithelial origin of myofibroblasts in kidney fibrosis Am J Pathol 2010 2 85 97 10.2353/ajpath.2010.090517 20008127 PMC2797872

[B83] Chawla LS Amdur RL Amodeo S Kimmel PL Palant CE The severity of acute kidney injury predicts progression to chronic kidney disease Kidney Int 2011 2 1361 1369 10.1038/ki.2011.42 21430640 PMC3257034

[B84] Grgic I Campanholle G Bijol V Wang C Sabbisetti VS Ichimura T Targeted proximal tubule injury triggers interstitial fibrosis and glomerulosclerosis Kidney Int 2012 2 172 183 10.1038/ki.2012.20 22437410 PMC3480325

[B85] Sagrinati C Netti GS Mazzinghi B Lazzeri E Liotta F Frosali F Isolation and characterization of multipotent progenitor cells from the Bowman’s capsule of adult human kidneys J Am Soc Nephrol 2006 2 2443 2456 10.1681/ASN.2006010089 16885410

[B86] Lazzeri E Crescioli C Ronconi E Mazzinghi B Sagrinati C Netti GS Regenerative potential of embryonic renal multipotent progenitors in acute renal failure J Am Soc Nephrol 2007 2 3128 3138 10.1681/ASN.2007020210 17978305

[B87] Ronconi E Sagrinati C Angelotti ML Lazzeri E Mazzinghi B Ballerini L Regeneration of glomerular podocytes by human renal progenitors J Am Soc Nephrol 2009 2 322 332 10.1681/ASN.2008070709 19092120 PMC2637058

[B88] Kriz W Podocyte hypertrophy mismatch and glomerular disease Nat Rev Nephrol 2012 2 618 619 10.1038/nrneph.2012.198 23007616

[B89] Petermann AT Pippin J Durvasula R Pichler R Hiromura K Monkawa T Mechanical stretch induces podocyte hypertrophy in vitro Kidney Int 2005 2 157 166 10.1111/j.1523-1755.2005.00066.x 15610239

[B90] Xu ZG Yoo TH Ryu DR Park HC Ha SK Han DS Angiotensin II receptor blocker inhibits p27^Kip1^ expression in glucose-stimulated podocytes and diabetic glomeruli Kidney Int 2005 2 944 952 10.1111/j.1523-1755.2005.00158.x 15698433

[B91] Osterby R Gundersen HJ Glomerular size and structure in diabetes mellitus. I. Early abnormalities Diabetologia 1975 2 225 229 10.1007/BF00422326 1149955

[B92] Gundersen HJ Osterby R Glomerular size and structure in diabetes mellitus. II. Late abnormalities Diabetologia 1977 2 43 38 10.1007/BF00996326 838202

[B93] Pabst R Sterzel RB Cell renewal of glomerular cell types in normal rats. An autoradiographic analysis Kidney Int 1983 2 626 631 10.1038/ki.1983.203 6663985

[B94] Appel D Kershaw DB Smeets B Yuan G Fuss A Frye B Recruitment of podocytes from glomerular parietal epithelial cells J Am Soc Nephrol 2009 2 333 343 10.1681/ASN.2008070795 19092119 PMC2637040

[B95] Bruno S Bussolati B Grange C Collino F de Cantogno LV Herrera MB Isolation and characterization of resident mesenchymal stem cells in human glomeruli Stem Cells Dev 2009 2 867 879 10.1089/scd.2008.0320 19579288

[B96] Swetha G Chandra V Phadnis S Bhonde R Glomerular parietal epithelial cells of adult murine kidney undergo EMT to generate cells with traits of renal progenitors J Cell Mol Med 2011 2 396 413 10.1111/j.1582-4934.2009.00937.x 19840197 PMC3822804

[B97] Smeets B Angelotti ML Rizzo P Dijkman H Lazzeri E Mooren F Renal progenitor cells contribute to hyperplastic lesions of podocytopathies and crescentic glomerulonephritis J Am Soc Nephrol 2009 2 2593 2603 10.1681/ASN.2009020132 19875807 PMC2794226

[B98] Smeets B Uhlig S Fuss A Mooren F Wetzels JFM Floege J Tracing the origin of glomerular extracapillary lesions from parietal epithelial cells J Am Soc Nephrol 2009 2 2604 2615 10.1681/ASN.2009010122 19917779 PMC2794233

[B99] Smeets B Moeller MJ Parietal epithelial cells and podocytes in glomerular diseases Semin Nephrol 2012 2 357 367 10.1016/j.semnephrol.2012.06.007 22958490

[B100] Lasagni L Ballerini L Angelotti ML Parente E Sagrinati C Mazzinghi B Notch activation differentially regulates renal progenitors proliferation and differentiation toward the podocyte lineage in glomerular disorders Stem Cells 2010 2 1673 1685 10.1002/stem.492 PMC2996085 20680961

[B101] Ruggenenti P Perna A Gherardi G Gaspari F Benini R Remuzzi G Renal function and requirement for dialysis in chronic nephropathy patients on long-term ramipril: REIN follow-up trial Lancet 1998 2 1252 1256 10.1016/S0140-6736(98)04433-X 9788454

[B102] Ruggenenti P Perna A Benini R Bertani T Zoccali C Maggiore Q In chronic nephropathies prolonged ACE inhibition can induce remission: dynamics of time-dependent changes in GFR J Am Soc Nephrol 1999 2 997 1006 10232685 10.1681/ASN.V105997

[B103] Wilmer WA Hebert LA Lewis EJ Rohde RD Whittier F Cattran D Remission of nephrotic syndrome in type 1 diabetes: long-term follow-up of patients in the captopril study Am J Kidney Dis 1999 2 308 314 10.1016/S0272-6386(99)70360-4 10430979

[B104] Macconi D Sangalli F Bonomelli M Conti S Condorelli L Gagliardini E Podocyte repopulation contributes to regression of glomerular injury induced by ACE inhibition Am J Pathol 2009 2 797 807 10.2353/ajpath.2009.080227 19164508 PMC2665741

[B105] Benigni A Morigi M Rizzo P Gagliardini E Rota C Abbate M Inhibiting angiotensin-converting enzyme promotes renal repair by limiting progenitor cell proliferation and restoring glomerular architecture Am J Pathol 2011 2 628 638 10.1016/j.ajpath.2011.04.003 21718676 PMC3157222

[B106] Oliver JA Lkinakis A Cheema FH Friedlander J Sampogna RV Martens TP Proliferation and migration of label-retaining cells of the kidney papilla J Am Soc Nephrol 2009 2 2315 2327 10.1681/ASN.2008111203 19762493 PMC2799170

[B107] Adams DC Oxburgh L The long-term label retaining population of the renal papilla arises through divergent regional growth of the kidney Am J Physiol Renal Physiol 2009 2 F809 F815 10.1152/ajprenal.90650.2008 19535568 PMC2739716

[B108] Patschan D Michurina T Shi HK Dolff S Brodsky SV Cohen-Gould L Normal distribution and medullary-to-cortical shift of Nestin-expressing cells in acute renal ischemia Kidney Int 2007 2 744 754 10.1038/sj.ki.5002102 17290297

[B109] Ward HH Romero E Welford A Pickett G Bacallao R Gattone VH II Adult human CD133/1+ kidney cells isolated from renal papilla integrate into developing kidney tubules Biochim Biophys Acta 1812 2 1344 1357 10.1016/j.bbadis.2011.01.010 PMC3166446 21255643

[B110] Song J Czerniak S Wang T Ying W Carlone DL Breault DT Characterization and fate of telomerase-expressing epithelial during kidney repair J Am Soc Nephrol 2011 2 2256 2265 10.1681/ASN.2011050447 22021716 PMC3250207

[B111] Kitamura S Yamasaki Y Kinomura M Sugaya T Sugiyama H Maeshima Y Establishment and characterization of renal progenitor like cells from S3 segment of nephron in rat adult kidney FASEB J 2005 2 1789 1797 10.1096/fj.05-3942com 16260649

[B112] Dekel B Zangi L Shezen E Reich-Zeliger S Eventov-Friedman S Katchman H Isolation and characterization of nontubular Sca-1+Lin- multipotent stem/progenitor cells from adult mouse kidney J Am Soc Nephrol 2006 2 3300 3314 10.1681/ASN.2005020195 17093069

[B113] Gupta S Verfaillie C Chmielewski D Kren S Eidman K Connaire J Isolation and characterization of kidney-derived stem cells J Am Soc Nephrol 2006 2 3028 3040 10.1681/ASN.2006030275 16988061

[B114] Lee PT Lin HH Jiang ST Lu PJ Chou KJ Fang HC Mouse kidney progenitor cells accelerate renal regeneration and prolong survival after ischemic injury Stem Cells 2010 2 573 584 20099318 10.1002/stem.310

[B115] Park HC Yasuda K Kuo MC Ni J Ratliff B Chander P Renal capsule as a stem cell niche Am J Physiol Renal Physiol 2010 2 F1254 F1262 10.1152/ajprenal.00406.2009 20200095 PMC2867407

[B116] Plotkin MD Goligorski MS Mesenchymal cells from adult kidney support angiogenesis and differentiate into multiple interstitial cell types including erythropoietin-producing fibroblasts Am J Renal Physiol 2006 2 F902 F912 10.1152/ajprenal.00396.2005 16622175

[B117] Chen J Park HC Addabbo F Ni J Pelger E Li H Plotkin M Kidney-derived mesenchymal stem cells contribute to vasculogenesis, angiogenesis and endothelial repair Kidney Int 2008 2 879 889 10.1038/ki.2008.304 18596729 PMC2782525

[B118] Asakura A Rudnicki MA Side population cells from diverse adult tissues are capable of in vitro hematopoietic differentiation Exp Hematol 2002 2 1339 1345 10.1016/S0301-472X(02)00954-2 12423688

[B119] Hishikawa K Marumo T Miura S Nakanishi A Matsuzaki Y Shibata K Musculin/MyoR is expressed in kidney side population cells and can regulate their function J Cell Biol 2005 2 921 928 10.1083/jcb.200412167 15967813 PMC2171631

[B120] Iwatani H Ito T Imai E Matsuzaki Y Suzuki A Yamato M Hematopoietic and nonhematopoietic potentials of Hoechstlow/side population cells isolated from adult rat kidney Kidney Int 2004 2 1604 1614 10.1111/j.1523-1755.2004.00561.x 15086898

[B121] Challen GA Bertoncello I Deane JA Ricardo SD Little MH Kidney side population reveals multilineage potential and renal functional capacity but also cellular heterogeneity J Am Soc Nephrol 2006 2 1896 1912 10.1681/ASN.2005111228 16707564

[B122] Addla SK Brown MD Hart CA Ramani VAC Clarke NW Characterization of the Hoechst 33342 side population from normal and malignant human renal epithelial cells Am J Physiol Renal Physiol 2008 2 F680 F687 10.1152/ajprenal.90286.2008 18614618 PMC2536866

[B123] Inowa T Hishikawa K Takeuchi T Kitamura T Fujita T Isolation and potential existence of side population cells in adult human kidney Int J Urol 2008 2 272 275 10.1111/j.1442-2042.2007.01984.x 18304230

[B124] Imai N Hishikawa K Marumo T Hirahashi J Inowa T Matsuzaki Y Inhibition of histone deacetylase activates side population cells in kidney and partially reverses chronic renal injury Stem Cells 2007 2 2469 2475 10.1634/stemcells.2007-0049 17641247

[B125] Deane JA Ricardo SD Emerging roles for primary cilia in epithelial repair Int Rev Cell Mol Biol 2012 2 169 193 22251562 10.1016/B978-0-12-394304-0.00011-7

[B126] Rodat-Despoix L Delmas P Ciliar functions in the nephron Pflugers Arch 2009 2 179 87 10.1007/s00424-008-0632-0 19153764

[B127] Saraga-Babic M Vukojevic K Bocina I Drnasin K Saraga M Ciliogenesis in normal human kidney development and post-natal life Pediatr Nephrol 2012 2 55 63 10.1007/s00467-011-1941-7 21688189

[B128] Romagnani P Lasagni L Remuzzi G Renal progenitors: an evolutionary conserved strategy for kidney regeneration Nat Rev Nephrol 2013 2 137 146 23338209 10.1038/nrneph.2012.290

[B129] Trounson A Thakar RG Lomax G Gibbons D Clinical trials for stem cell therapies BMC Medicine 2011 2 52 10.1186/1741-7015-9-52 21569277 PMC3098796

[B130] Zeisberg M Kalluri R Reversal of experimental renal fibrosis by BMP7 provides insights into novel therapeutic strategies for chronic kidney disease Pediatr Nephrol 2008 2 1395 1398 10.1007/s00467-008-0818-x 18446379

[B131] Choudhury D Levi M Kidney aging—inevitable or preventable? Nat Rev Nephrol 2011 2 706 717 10.1038/nrneph.2011.104 21826079

[B132] McCreight CE Sulkin NM Cellular proliferation in the kidneys of young and senile rats following unilateral nephrectomy J Gerontol 1959 2 440 443 10.1093/geronj/14.4.440

[B133] Yang HC Rossini M Ma LJ Zuo Y Ma J Fogo AB Cells derived from young bone marrow alleviate renal aging J Am Soc Nephrol 2011 2 2028 2036 10.1681/ASN.2010090982 21965376 PMC3231782

